# Postsynaptic Assembly: A Role for Wnt Signaling

**DOI:** 10.1002/dneu.22138

**Published:** 2013-10-07

**Authors:** Eleanna Stamatakou, Patricia C Salinas

**Affiliations:** Department of Cell and Developmental Biology, University College London,London WC1E 6BT, United Kingdom

**Keywords:** neuromuscular junction, excitatory and inhibitory synapse, dendritic spines, synaptic plasticity

## Abstract

Synapse formation requires the coordinated formation of the presynaptic terminal, containing the machinery for neurotransmitter release, and the postsynaptic side that possesses the machinery for neurotransmitter reception. For coordinated pre- and postsynaptic assembly signals across the synapse are required. Wnt secreted proteins are well-known synaptogenic factors that promote the recruitment of presynaptic components in diverse organisms. However, recent studies demonstrate that Wnts act directly onto the postsynaptic side at both central and peripheral synapses to promote postsynaptic development and synaptic strength. This review focuses on the role of Wnts in postsynaptic development at central synapses and the neuromuscular junction. © 2013 The Authors. Developmental Neurobiology Published by Wiley Periodicals, Inc. Develop Neurobiol 74: 818–827, 2014

## INTRODUCTION

The formation of neuronal networks is a complex process that requires the precise and coordinated activation of several signaling pathways. Neurons extend their axons in search for their synaptic targets. Once axons have reached their synaptic partners, begin to assemble synapses by the recruitment of hundreds of pre- and postsynaptic components (Sanes and Lichtman, 2001; McAllister, 2007; Sheng and Kim, 2011). The interplay between secreted factors, adhesion molecules, and intracellular signaling molecules determine the formation of different types of synapses, such as excitatory and inhibitory.

The postsynaptic side plays a crucial role in synaptic signaling and plasticity. Its structure depends on the type of synapse. In the central nervous system, most excitatory synapses are formed onto dendritic spines, which are actin-rich structures protruding from the dendritic shaft. Spines are composed of a head that contains the components important for synaptic signaling, and a neck connecting the spine head to the dendritic shaft resulting in the strict compartmentalization of synaptic signaling between individual synapses (Bourne and Harris, 2008; Rochefort and Konnerth, 2012). Inhibitory synapses, in contrast, are formed directly on the dendritic shaft. In the periphery, the neuromuscular synapse has a completely different structure as the postsynaptic side is formed on a muscle rather than a neuron. These structural characteristics suggest that different signaling mechanisms might control the assembly of the postsynaptic apparatus of these different types of synapses.

Wnt signaling has been shown to modulate the formation and the function of different types of synapses. Wnts are a large family (19 members in humans) of secreted lipid-modified glycoproteins that are highly evolutionary conserved. The term Wnt derives from of the *Drosophila* gene *wingless* (*wg*) and the vertebrate gene *Int-1* (Nusse et al., 1991). The role of Wg in early embryonic patterning was characterized almost 40 years ago (Sharma and Chopra, 1976). Since then, studies in different organisms have demonstrated a crucial role for Wnt signaling in several biological processes ranging from stem cell self-renewal to cell fate decisions, tissue polarization, and cellular homeostasis (Arwert et al., 2012; Merrill, 2012; Regard et al., 2012; von Maltzahn et al., 2012; Anastas and Moon, 2013). In the nervous system, Wnts play an important role in neuronal connectivity by regulating axon guidance, dendritic arborization, and synapse formation (Ciani and Salinas, 2005; Fradkin et al., 2005; Salinas and Zou, 2008; Inestrosa and Arenas, 2010; Budnik and Salinas, 2011; Koles and Budnik, 2012; Mulligan and Cheyette, 2012; Park and Shen, 2012).

During the last two decades great progress has been made in elucidating the signaling pathways activated by Wnt proteins, broadly categorized as canonical and noncanonical pathways (Veeman et al., 2003; Kohn and Moon, 2005; Gordon and Nusse, 2006; Salinas, 2007; van Amerongen and Nusse, 2009; Niehrs, 2012; Behrens, 2013). The best understood cascade is the β-catenin (or canonical) pathway where binding of Wnts to Frizzled receptors (Fz) and their coreceptors LRP5/6 activates gene expression. Downstream of these receptors is Dishevelled (Dvl), a scaffold protein, which is required for all Wnt signaling cascades. Activation of Dvl leads to the inhibition of the serine/threonine kinase Gsk3β and the subsequent accumulation of the cytoplasmic protein β-catenin, which translocates to the nucleus to promote TCF/LEF-mediated transcription (Gordon and Nusse, 2006). In addition, a divergent canonical pathway, which does not signal to the nucleus but still requires Gsk3β inhibition, regulates cytoskeletal changes (Salinas, 2007). The next well-known Wnt cascade is the planar cell polarity pathway (PCP), which through Fz receptors and Dvl leads to the activation of small Rho-GTPases to control cell and tissue polarity (Veeman et al., 2003). The third pathway is the calcium-signaling cascade, which results in elevation of intracellular calcium and activation of calcium/calmodulin dependent protein kinase II (CaMKII) (Kohn and Moon, 2005). At the *Drosophila* neuromuscular junction (NMJ), an additional pathway has been described (Mathew et al., 2005). This pathway is activated by the binding of Wg, which is released from the presynaptic motor neuron, to the Frizzled 2 (Dfz2) receptor on the postsynaptic muscle cell. Upon Wg binding, the C-terminus of the DFz2 receptor is cleaved and transported to the nucleus (Mathew et al., 2005). Thus, Wnts can activate different pathways and elicit a variety on responses depending on the cellular context.

### Wnts at the Synapse

Expression of Wnts, their receptors and signaling components in the nervous system during the formation of neuronal connections suggested that Wnt signaling plays a role in different aspects of neuronal circuit assembly and function in the adult. The role of Wnt signaling in synapse formation was first demonstrated in cultured neurons (Lucas and Salinas, 1997). Wnt7a, which is released from cerebellar granule cells, stimulates the presynaptic differentiation of mossy fiber terminals and the accumulation of the presynaptic marker Synapsin I (Hall et al., 2000). Importantly, loss of function of Wnt7a results in defects at the accumulation of presynaptic components at mossy fiber terminals, suggesting that Wnt7a acts as a retrograde signal from granule cells to mossy fiber axons (Hall et al., 2000). Since then, great progress has been made in elucidating the role of Wnts in synaptogenesis and dissecting the signaling pathways activated at the synapse. Wnt proteins regulate synaptogenesis in both vertebrates and invertebrates by acting as pro- or antisynaptogenic factors (Inestrosa and Arenas, 2010; Budnik and Salinas, 2011; Sahores and Salinas, 2011; Koles and Budnik, 2012; Park and Shen, 2012).

Although initial studies were focused on the role of Wnts on presynaptic assembly, recent studies have demonstrated that Wnts directly signal to the postsynaptic side at both central and peripheral synapses to promote postsynaptic development and synaptic strength (Mathew et al., 2005; Henriquez et al., 2008; Ciani et al., 2011; Jensen et al., 2012). Here we discuss the recent advances in understanding the role of different Wnt signaling pathways in the assembly of the postsynaptic structures by focusing our attention to synapses in the central and peripheral system in different model organisms.

## CENTRAL SYNAPSES

### Excitatory Synapses

In the central nervous system, most excitatory inputs are formed on dendritic spines. These structures emerge from filopodia, which get converted to mushroom-like structures through changes in the structure and dynamics of the actin cytoskeleton (Hotulainen and Hoogenraad, 2010; Svitkina et al., 2010; Penzes and Cahill, 2012). Concomitant with these structural changes, postsynaptic components are recruited to the synapse. Glutamate receptors are enriched at the postsynaptic density (PSD) through the interaction with scaffold proteins such as PSD95, Shank and Homer (McAllister, 2007; Feng and Zhang, 2009). In addition, signaling molecules such as CaMKII, small GTPases, and actin-binding proteins are also recruited (Sheng and Kim, 2011). Despite extensive studies on the structure of excitatory synapses, little is known about the mechanisms that trigger the assembly of the postsynapse and how this relates with the recruitment of synaptic components on the adjacent presynaptic terminal.

Three different members of the Wnt family have been shown to induce postsynaptic formation: Wnt7a, Wnt5a and more recently Wnt2 (Farias et al., 2009; Varela-Nallar et al., 2010; Ciani et al., 2011; Hiester et al., 2013). However, since Wnts play a crucial role in presynaptic assembly at central synapses (Lucas and Salinas, 1997; Hall et al., 2000; Ahmad-Annuar et al., 2006; Varela-Nallar et al., 2009; Paganoni et al., 2010; Sahores et al., 2010), this is raising the question as to whether the effect on the postsynaptic side is indirectly following the assembly of the presynaptic bouton.

**Figure 1:**
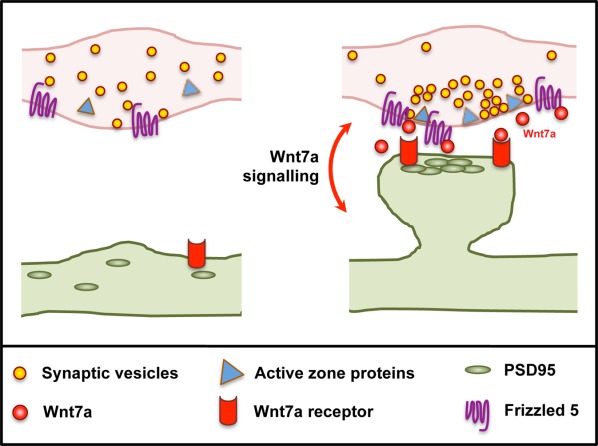
Wnt7a acts bi-directionally to promote synaptic assembly. On axons, Wnt7a binds to Fz5 receptors and induces the recruitment of presynaptic components, including synaptic vesicles and active zone proteins. On dendrites, Wnt7a promotes PSD95 clustering, and spine formation and growth, through an unidentified receptor.

A direct role of Wnt signaling in the postsynaptic differentiation of excitatory synapses was demonstrated by gain and loss of function studies of Dvl1, a scaffold protein and a key component of the Wnt pathway (Ciani et al., 2011). While gain of function of Dvl1 mimics the effect of Wnt7a on spine morphogenesis, loss of function studies provided evidence that Dvl1 is required on dendrites for Wnt7a to regulate spine formation (Ciani et al., 2011). These findings demonstrate that Wnt signaling is important for the coordinated development of both sides of the synapse (Fig. 1).

**Figure 2:**
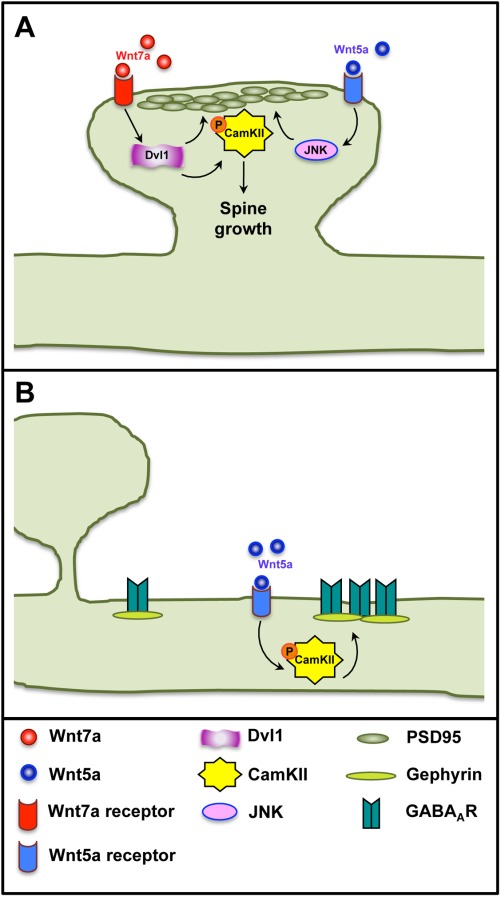
Wnt5a and Wnt7a regulate the assembly of central synapses. (A) Wnt7a and Wnt5a induce the formation of postsynaptic structures at excitatory synapses. Wnt7a, through Dvl1 and CaMKII, induces the recruitment of PSD95 and increases spine growth. In contrast, Wnt5a induces PSD95 clustering via JNK. (B) Wnt5a also promotes the formation of inhibitory synapses by inducing the clustering of GABA_A_ receptors through CaMKII at inhibitory synapses.

Consistent with the effect of Wnt7a signaling on spine formation and growth, Wnt7a increases the frequency and the amplitude of miniature excitatory postsynaptic currents (mEPSCs). Importantly, double mutant mice for *Wnt7a* and *Dvl1* exhibit strong deficits in spine morphogenesis and synaptic strength in the hippocampus (Ciani et al., 2011). Postsynaptic Wnt7a signaling promotes PSD95 clustering and the rapid local activation of CaMKII within dendritic spines (Fig. 2) (Ciani et al., 2011). Notably, CaMKII is required for Wnt7a-mediated spine growth and increased synaptic strength. These findings clearly demonstrate a novel role for Wnt7a signaling in promoting postsynaptic differentiation and maturation through CaMKII, a key molecule involved in synaptic plasticity.

Studies on Wnt5a, another Wnt family member, suggest its role in excitatory synapse formation, but the findings are controversial. Wnt5a induces postsynaptic differentiation at excitatory synapses in hippocampal neurons by rapidly inducing the clustering of PSD95 through a noncanonical pathway that requires JNK activation (Fig. 2) (Farias et al., 2009). Although it has been reported that Wnt5a promotes spine morphogenesis through a calcium-dependent signaling pathway (Varela-Nallar et al., 2010), other studies indicate that Wnt5a does not affect spine formation (Farias et al., 2009; Cerpa et al., 2011). Further analyses including *in vivo* loss of function studies might resolve these apparent conflicting results. Intriguingly, it has been shown that short exposure to Wnt5a does not affect presynaptic assembly, whereas other studies have reported that Wnt5a promotes or even inhibits the clustering of presynaptic components (Cerpa et al., 2008; Davis et al., 2008; Farias et al., 2009; Paganoni et al., 2010; Cerpa et al., 2011; Varela-Nallar et al., 2012). Moreover, the frequency of mEPSCs is unaffected by Wnt5a (Cerpa et al., 2011). Therefore, it is currently unclear whether Wnt5a is a synaptogenic factor or a postsynaptic modulator.

In cortical neurons, exposure to the Wnt antagonist Sfrp1 results in fewer and smaller spines, suggesting a role for endogenous Wnts in spine morphogenesis in the cortex (Hiester et al., 2013). Indeed, Wnt2, which is expressed in several cortical areas, promotes spine morphogenesis (Hiester et al., 2013). Although the pathway activated by Wnt2 remains to be determined, studies on Wnt7a and Wnt5a demonstrate that different signaling pathways act on dendrites to promote postsynaptic assembly.

### Inhibitory Synapses

Synapses containing γ-aminobutyric acid (GABA) receptors represent the main inhibitory synapses in the central nervous system. Inhibitory synapses are located on the cell body and at the shaft of proximal dendritic branches. At these synapses, Gephyrin, the main scaffolding protein, regulates the clustering of GABA_A_ receptors (Fritschy et al., 2008, 2012). Although several signaling molecules have been shown to promote the assembly of inhibitory synapses (Takayama, 2005; Lu et al., 2009; Luscher et al., 2011), the molecular mechanisms remain poorly understood.

In addition to its role in excitatory synapses, Wnt5a modulates inhibitory synapses by increasing the surface localization and clustering of GABA_A_ receptors without affecting their total levels (Cuitino et al., 2010). This is accompanied by an increase in the amplitude, but not frequency, of miniature inhibitory postsynaptic currents (mIPSCs) and increased evoked inhibitory postsynaptic currents (eIPSCs). This effect of Wnt5a is mediated by CaMK signaling (Fig. 2) (Cuitino et al., 2010). Intriguingly, Wnt5a does not affect the clustering of Gephyrin despite affecting the clustering of GABA_A_Rs. Together these findings suggest that Wnt5a promotes the localization of GABA receptors without affecting the number of inhibitory synapses.

In sharp contrast with Wnt5a, Wnt7a has no effect on inhibitory synapses. Gain of function on cultured neurons demonstrates that Wnt7a does not affect the formation of inhibitory synapses nor does affect GABA-mediated mIPSCs (Ciani et al., 2011). Therefore, Wnt7a specifically promotes the formation of excitatory synapses, thus affecting the ratio of excitatory/inhibitory inputs. These results suggest that Wnt7a signaling could serve as a target for the treatment of neurological disorders where an imbalance between excitatory and inhibitory synapses is observed, such as autism and epilepsy.

## THE NEUROMUSCULAR JUNCTION

### Glutamatergic Synapses (*Drosophila*)

**Figure 3:**
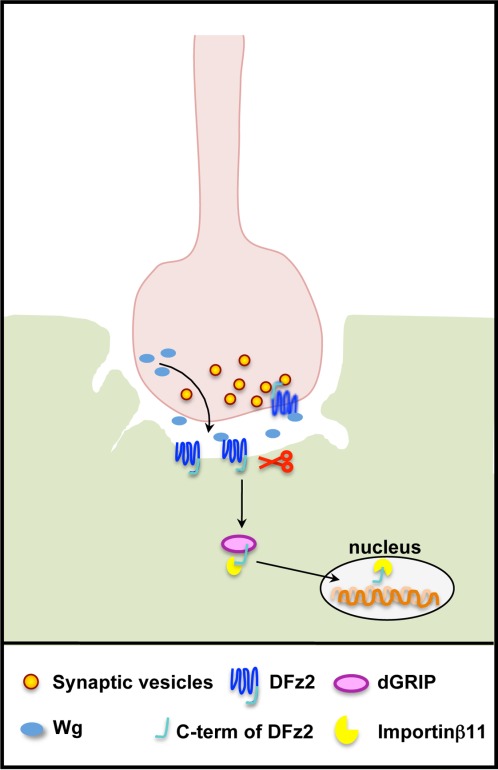
At the *Drosophila* NMJ, Wg activates a pathway that requires the processing of the Wnt receptor DFz2. Wg, secreted from presynaptic boutons, binds to DFz2 present in both sides of the synapse. In muscle cells, Wg binding to DFz2 triggers the cleavage and nuclear translocation of the C-terminus tail of this receptor. dGRIP and importinβ11 are required for the trafficking of the cleaved tail of DFz2 to the nucleus.

A synaptogenic role of Wnt signaling in the NMJ development was fist demonstrated in Drosophila. Wingless (Wg), the *Drosophila* Wnt1 homolog, is released by glutamatergic motor neurons innervating the body wall muscles of the fly larvae. Loss of function studies have demonstrated that Wg is required for both pre- and postsynaptic development (Packard et al., 2002). *wg* mutant flies display abnormal PSD morphology and mislocalized glutamate receptors. In addition, several boutons lack proper postsynaptic structures (ghost boutons) (Packard et al., 2002; Ataman et al., 2008; Mosca and Schwarz, 2010). The action of Wg is mediated through the DFz2 receptor, located at both sides of the synapse. At the postsynaptic side, binding of Wg to DFz2 promotes the cleavage of the cytoplasmic C-terminus tail of the receptor and its translocation to the nucleus in an dGRIP- and importinβ11-dependent manner (Fig. 3) (Mathew et al., 2005; Ataman et al., 2006, 2008; Mosca and Schwarz, 2010). Disruption of this pathway leads to aberrant pre- and postsynaptic structures (Ataman et al., 2006). Given that the cleavage of DFz2 on the postsynaptic muscle results to presynaptic defects, these studies suggest that this signaling pathway leads to the release of a retrograde signal that acts presynaptically. Thus, Wg signaling is crucial for the functional matching between pre- and postsynaptic structures in the *Drosophila* NMJ. However, further studies are needed to elucidate the mechanisms that control the clustering of glutamatergic receptors and the precise role of the DFz2 nuclear import pathway in postsynaptic differentiation.

### Cholinergic Synapses

In vertebrates, the NMJ was the first well-studied synapse due to its large size and accessibility. During synaptogenesis, motor neuron axons innervate the central region of the muscle where small clusters of acetylcholine receptors (AChRs) are present. Interestingly, these AChR clusters occur even before muscle innervation, through a process called “prepatterning” (Sanes and Lichtman, 2001; Lomo, 2003; Witzemann, 2006) and these clusters are called “aneural.” Agrin, a secreted proteoglycan released by motorneuron axons, promotes the formation of large and stable AChR clusters in perfect apposition to presynaptic boutons. In *Agrin* mutant mice, AChR prepatterning is normal, but the formation of large AChR clusters in a opposition to motor neuron terminals is highly defected (Gautam et al., 1996; Burgess et al., 1999). In contrast, muscle-specific kinase (MuSK) and low density lipoprotein receptor-related protein 4 (LRP4), two muscle-specific receptors for Agrin, are required for both prepatterning and Agrin-mediated formation of AChR clusters (DeChiara et al., 1996; Glass et al., 1996; Yang et al., 2001; Zhang et al., 2004; Weatherbee et al., 2006; Kim et al., 2008; Zhang et al., 2008). These findings provided initial evidence for the presence of addition factors that act through MuSK and LRP4 to regulate prepatterning.

#### Wnts on AChR Prepatterning

Studies at the zebrafish NMJ have demonstrated that Wnt signaling contributes to prepatterning of AChRs. Wnt11r binds to MuSK and is required for the formation of aneural clusters (Jing et al., 2009). However, these defects that are observed before the arrival of motor axons do not seem to affect the formation of the mature NMJ. In higher vertebrates, Wnt4, which also binds to MuSK plays a role in prepatterning. *Wnt4* mutant mice exhibit a significant reduction in the number of aneural AChR clusters (Strochlic et al., 2012). These studies demonstrate that two distinct ligands for MuSK, Wnt11r, and Wnt4, regulate the formation of aneural AChR clusters.

#### Wnt Signaling During Postsynaptic Development of the Vertebrate NMJ

Wnt signaling was first implicated in the proper assembly of the vertebrate NMJ by studies on Dvl1. This scaffold protein directly interacts with MuSK through its DEP domain (Luo et al., 2002). This binding is crucial for Agrin-mediated AChR clustering and NMJ synaptic transmission. Agrin induces AChR clustering through PAK1 activation in a Dvl1-dependent manner (Luo et al., 2002). Although gain of function of Dvl1 is not sufficient to induce AChR clustering, expression of a Dvl1 mutant that lacks the DEP domain blocks Agrin-mediated AChR clustering and PAK1 activation (Luo et al., 2002). Thus, Dvl1 is required for Agrin function at the NMJ. Consistent with this conclusion, *Dvl1* null mice display abnormal AChR distribution in the diaphragm (Henriquez et al., 2008).

**Figure 4:**
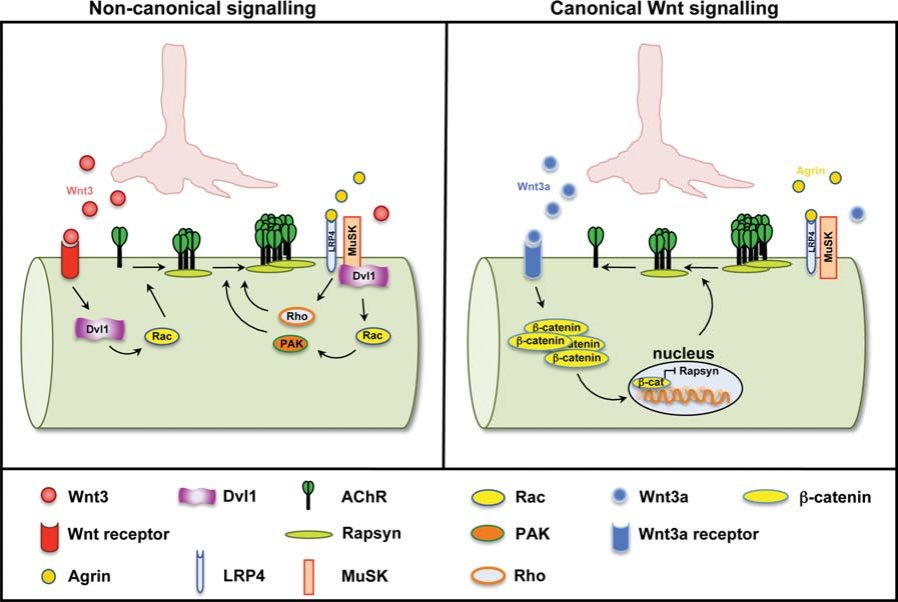
Canonical and non-canonical Wnt pathways exert opposite effects on the formation of the vertebrate NMJ. The noncanonical Wnt cascade (left panel) has a positive role on postsynaptic development. Wnt3 activates Rac1 and induces the formation of AChR microclusters, which are then converted to stable and large clusters in the presence of Agrin. In contrast, activation of the canonical Wnt pathway (right panel) has a negative effect on NMJ formation. Wnt3a, through β-catenin, inhibits the expression of Rapsyn resulting in the dispersal of AChR clusters.

A direct role of Wnt ligands in postsynaptic assembly of the vertebrate NMJ was demonstrated by gain and loss of function experiments in chick embryos and in cultured myotubes. Implantation of cells expressing the Wnt antagonist Sfrp1 into the chick wing, at the time when motorneurons begin to innervate muscles results in a significant reduction of AChR clustering (Henriquez et al., 2008). This finding demonstrates that endogenous Wnts are required for postsynaptic assembly. Wnt3, which is expressed in the mouse spinal cord when motor neurons innervate muscle cells at the limb level (Krylova et al., 2002), rapidly induces the formation of unstable AChR microclusters. In the presence of Agrin, these microclusters become large and stable. Formation of microclusters by Wnt3 requires Rac1 activation (Henriquez et al., 2008). However, Agrin also induces in the activation of Rho, which is crucial for the subsequent conversion of these microclusters into stable AChR clusters (Fig. 4; left panel) (Weston et al., 2000, 2003). Together these studies suggest that Wnt3 collaborates with Agrin to promote the formation of large and stable AchR clusters, a hallmark of postsynaptic differentiation at the NMJ.

Another Wnt factor that regulates AChR clustering is Wnt4 (Strochlic et al., 2012). However, the phenotype elicited by Wnt4 is complex. While in cultured myotubes, Wnt4 increases the number of AChR clusters in the absence of Agrin, the *Wnt4* mutant mouse exhibits normal postsynaptic structures in limb muscles and in the diaphragm, where Wnt4 is expressed (Strochlic et al., 2012). However, muscle innervation is significantly affected. These results suggest that *in vivo* Wnt4 regulates neuronal innervation without affecting postsynaptic assembly. Although other Wnts have been shown to bind to MuSK and to promote AChR clustering in cultured myotubes (Zhang et al., 2012), their *in vivo* role remains to be established.

In contrast to Wnt3, Wnt4, and other Wnts, activation of the canonical pathway by Wnt3a results in the inhibition of NMJ development. Wnt3a reduces Agrin-mediated AChR clustering by inhibiting the expression of Rapsyn, a scaffold protein crucial for the anchoring of AChRs. This effect is β-catenin-dependent (Fig. 4; right panel), but TCF-independent, and is blocked by the canonical Wnt signaling inhibitor Dkk1 (Wang et al., 2008). Importantly, *in vivo* gain of function of Wnt3a or β-catenin in mouse limb muscles leads to the disassembly of the postsynaptic apparatus (Wang et al., 2008). Moreover, β-catenin stabilization in the diaphragm results in a wider distribution of AChR clusters (Liu et al., 2012), as observed in the *Dvl1* or *Agrin* mutant mice (Gautam et al., 1996; Henriquez et al., 2008). In contrast, β-catenin null muscles possess larger AChRs clusters and increased Rapsyn levels (Li et al., 2008; Wang et al., 2008). Together these results demonstrate that canonical Wnt signaling is a negative regulator of NMJ development and suggest that a proper balance between canonical and noncanonical Wnt signaling might modulate the size of the NMJ. Further studies are required to elucidate the mechanisms that regulate this balance during the assembly, growth, and elimination of the NMJ.

LRP4, a single pass receptor highly homologs to the Wnt coreceptors LRP5/6 and the *Drosophila* homolog Arrow, plays a crucial role in the formation of the NMJ. LRP4 mutant mice exhibit profound defects in the formation of AChR clusters (Weatherbee et al., 2006), similar to those observed in the MuSK mutant (DeChiara et al., 1996; Yang et al., 2001). Even though the extracellular domain of LRP4 is very similar to the domain in LRP5/6 and Arrow (He et al., 2004), the binding of Wnts to LRP4 has not been reported. Given that the canonical pathway has an inhibitory effect on NMJ formation, it is intriguing to speculate that “canonical Wnts” might inhibit synapse formation or restrict its growth by interfering with the binding of Agrin to the LRP4. Further studies are required to explore this possibility.

#### Wnts Regulate Postsynaptic Invertebrate NMJ Development

Wnt signaling is also important for AChR surface expression at invertebrate synapses. In *C. elegans*, the NMJ contains two classes of AChRs, the levamisole-sensite and the nicotine-sensitive receptors (L-AChR and N-AChR, respectively). The synaptic localization of surface N-AChRs is highly dependent on the CAM-1 receptor, a member of the Ror family tyrosine kinase receptors (RTK) that function as Wnt receptors (Francis et al., 2005). CAM-1 contains an extracellular cysteine-rich domain (CRD), similar to the CRD domain in Fz receptors, which are required for Wnt binding. These findings suggest that N-AChR surface levels are modulated by Wnt signaling. Indeed, *in vivo* studies using loss of function of Wnt signaling components and muscle-specific rescue experiments demonstrate that Wnt signaling is required for proper synaptic localization of N-AChRs and synaptic strength (Jensen et al., 2012). CWN-2 (a Wnt protein), through LIN-17 (a Frizzled receptor) and DHS-1 (a Dvl homolog), controls postsynaptic strength (Jensen et al., 2012). Loss of function of these components leads to movement defects and aberrant postsynaptic currents, without affecting presynaptic function. Importantly, expression of LIN-17 and DSH-1 in muscles, but not in neurons, is sufficient to rescue the defects observed in these mutants (Jensen et al., 2012). These effects are accompanied with changes in the surface localization of N-AChRs (Jensen et al., 2012). As observed at the vertebrate NMJ, in the worm, Wnt signaling promotes the postsynaptic localization of AChRs, a key requirement for postsynaptic assembly.

## CONCLUDING REMARKS

In this review, we have discussed the contribution of distinct Wnt signaling pathways in the formation of different types of synapses. Interestingly, activation of the same signaling pathway can lead to the formation of different synapse types. For example, Wnt7a signaling activates CaMKII to promote spine growth, whereas Wnt5a also through CaMKII increases the clustering of synaptic components at inhibitory synapses. These findings suggest that different receptor complexes might contribute to these very distinct outcomes. Future studies on the localization and activation of Wnt receptors will shed new light into the specific molecular events that lead to the assembly of postsynaptic structures at different types of synapses.

Recent studies show that the surface localization of Fz5 at synapses is regulated by neuronal activity. Moreover, endogenous Wnts are required for the activity-induced trafficking of Fz5. Importantly, blockade of endogenous Wnts completely abolishes activity-mediated synapse formation in hippocampal neurons. These results suggest the exciting possibility that Wnts play a central role in experience-dependent synapse formation including synaptic changes associated with learning and memory.

The formation of central excitatory synapses requires the coordinated recruitment of postsynaptic components and also modification of the actin cytoskeleton to form dendritic spines. Some of the very upstream molecules regulated by Wnts have been identified. However, it is unclear how Wnt signaling modulates the actin cytoskeleton at the post-synapse. Given that specific patterns of neuronal activity modulate the formation and morphology of excitatory synapses, studies on Wnts will also elucidate the molecular mechanisms that regulate synaptic plasticity.
